# Diagnosing Porphyria in a Female Patient With Diffuse Pelvic Pain: A Case Study

**DOI:** 10.7759/cureus.74839

**Published:** 2024-11-30

**Authors:** Lauren N Hoffpauir, Rob Olexo, Hiliary Hamric

**Affiliations:** 1 Family Medicine, West Virginia School of Osteopathic Medicine, Lewisburg, USA; 2 Pediatrics, West Virginia School of Osteopathic Medicine, Lewisburg, USA

**Keywords:** acute intermittent porphyria, acute porphyria, altered mental state, lower abdominal pain, young women with abdominal pain

## Abstract

Porphyria is a rare and often underdiagnosed metabolic disorder that leads to abdominal pain, pelvic pain, changes in neurological states, and digestive issues due to a buildup of porphyrins in the body. Diagnosis can be especially difficult in young women, where symptoms of porphyria often overlap with gynecological conditions. We present a case of a 37-year-old female patient who was experiencing persistent lower abdominal and pelvic pain, brain fog and confusion, and a rash after long sun exposure. Despite extensive evaluation from her gynecologist, including transvaginal ultrasound and pap smears, no abnormalities were found. Further evaluation performed by her primary care physician showed elevated porphyrin levels, suggesting a possible diagnosis of either hereditary coproporphyria or acute intermittent porphyria. After implementing a low-protein diet, the patient reported significant pain relief, which further supports a diagnosis of porphyria. This case highlights not only the challenges of diagnosing porphyria in young women but also the value of considering metabolic disorders in a preliminary differential diagnosis. Diagnostic laboratory testing of urine and stool remains the gold standard. However, advances in genetic testing have shown to be more conclusive, yet these tests are costly.

## Introduction

Porphyria is a rare condition affecting less than 200,000 people within the United States [1]. Porphyria causes significant abdominal pain due to the buildup of porphyrins in the body, which are needed for hemoglobin synthesis [2]. With eight subclasses, porphyria can have differing manifestations, most of which include abdominal pain that may radiate to the back or lower extremities, while most cases additionally cause digestive issues, changes to the color of urine, and neurological issues [2]. The multiple presentations and varying degrees of severity make porphyria cases difficult to diagnose. Furthermore, definitively identifying which type of porphyria is present requires urine, stool, and genetic testing for specific enzymatic precursors or mutations [3]. Porphyria can be especially difficult to diagnose in young women because symptoms mimic menstruation and several other gynecological etiologies. The hormone levels, such as estrogen and progesterone that rise and fall significantly during menstruation, also induce porphyrin enzymatic precursors [4]. Consequently, confusion between the presentation of porphyria and gynecological issues leads to underdiagnosed cases of porphyria in young women of childbearing age.

## Case presentation

A 37-year-old Caucasian female patient presented to the office with lower abdominal and pelvic pain for the past six months. She stated that the pain was not positional, not related to menstrual cycles, and that it was consistently dull but increased in severity periodically. Multiple recent changes in diet and medications have caused episodes of pain so intense that the patient was unable to move. She stated that her oral birth control was discontinued years ago due to changes in mental status, irritation, and fatigue. The lower abdominal pain caused the patient to visit her gynecologist with lower abdominal pain; the patient underwent a Pap smear and transvaginal and transabdominal ultrasounds, which demonstrated no findings. Gynecological testing was performed under the direction of another physician, and ultrasound imaging was not available in the patient's chart. The gynecologist placed the patient on Macrobid for a possible urinary tract infection, which caused her significant abdominal pain and mental status change.

The pain continued to persist, causing the patient to present to her primary care physician (PCP), who referred the patient to the local hospital for a CT scan of the abdomen and pelvis. The radiology report sent to the PCP was also negative for findings; however, no radiology images were sent with the report.

Her daughter, age 16, was recently diagnosed with advanced celiac disease. Following her daughter’s diagnosis, the family began consuming more red meats and less carbohydrates. The patient’s pain became excruciating after eating a small steak for dinner and again several days later when she ate a cheeseburger. Upon further questioning, she reveals that she has been suffering from bouts of brain fog, where it is difficult to recall specifics about the day’s events or her purpose for entering a room. She also began a car detailing business, which caused her to be in the sun for hours. The patient broke out into a pustular, burning rash but attributed this to chemical exposure.

Urine was collected at her next visit to her PCP and sent for random quantitative total porphyrin laboratory testing. Results demonstrated uroporphyrins, hexacarbonyl, and pentacarbonyl levels within the normal range (normal: 0-2.0 ug/L). Additional urine testing showed a heptacarboxyl level of 4.0 ug/L (normal: 0-2.0 ug/L), a coproporphyrin I level of 39.0 ug/L (normal: 0-15.0 ug/L), and a coproporphyrin III level of 71.0 ug/L (normal: 0-49.0 ug/L), all of which are double the upper limits of normal. Concern for heavy metals indicated the need for serum lead levels, showing an undetectable range (normal: <1.0 ug/dL). A stool sample and serum porphyria samples were collected, both of which contained undetectable levels of porphyria. When asked, the patient stated that she had independently researched the disease and started her new “low protein diet” before the samples were collected. While this is not ideal for diagnostic purposes, she reports that her discomfort has completely subsided, indicating that porphyria was the cause of the initial abdominal pain.

The patient was consulted on possible treatment options for the disease process, including available medications. Conservative treatment with a high-carbohydrate/low-protein diet was shown to be beneficial for this patient within days of starting the diet, and she reported no further confusion or abdominal pain. 

## Discussion

There are two major classes of porphyria: acute porphyria and cutaneous types [1]. Most types of porphyria are inherited in an autosomal dominant pattern, although some subclasses have been found to be autosomal recessive [2]. Acute porphyrins most commonly present with abdominal, chest, and lower extremity pains, muscle tingling, discolored urine, and neurological issues such as confusion, seizures, and heart palpitations [2]. The wide range of symptoms make diagnosing porphyria difficult. In this patient’s case, occasional brain fogging, and pelvic pain were the only symptoms. Of the subclasses of porphyria, it is known that hereditary coproporphyria (HCP) typically has fewer symptoms than other types [1]. The immediate differentials after considering her negative gynecological exam included, but were not limited to endometriosis, celiac disease, and kidney stones. However, the neurological symptoms promoted a total porphyrin urinalysis to rule out porphyria. This patient’s results were most compelling for either a diagnosis of HCP, due to her elevated levels of coproporphyrin I and coproporphyrin III levels, or acute intermittent porphyria (AIP), which was unable to be ruled out as a possible cause of elevated urine porphyria. To further differentiate AIP from HCP, fecal porphyria levels were obtained. However, at the time of fecal collection, the patient had already started her low-protein diet, which likely altered the fecal porphyria levels, leading to an inconclusive result. 

**Figure 1:**
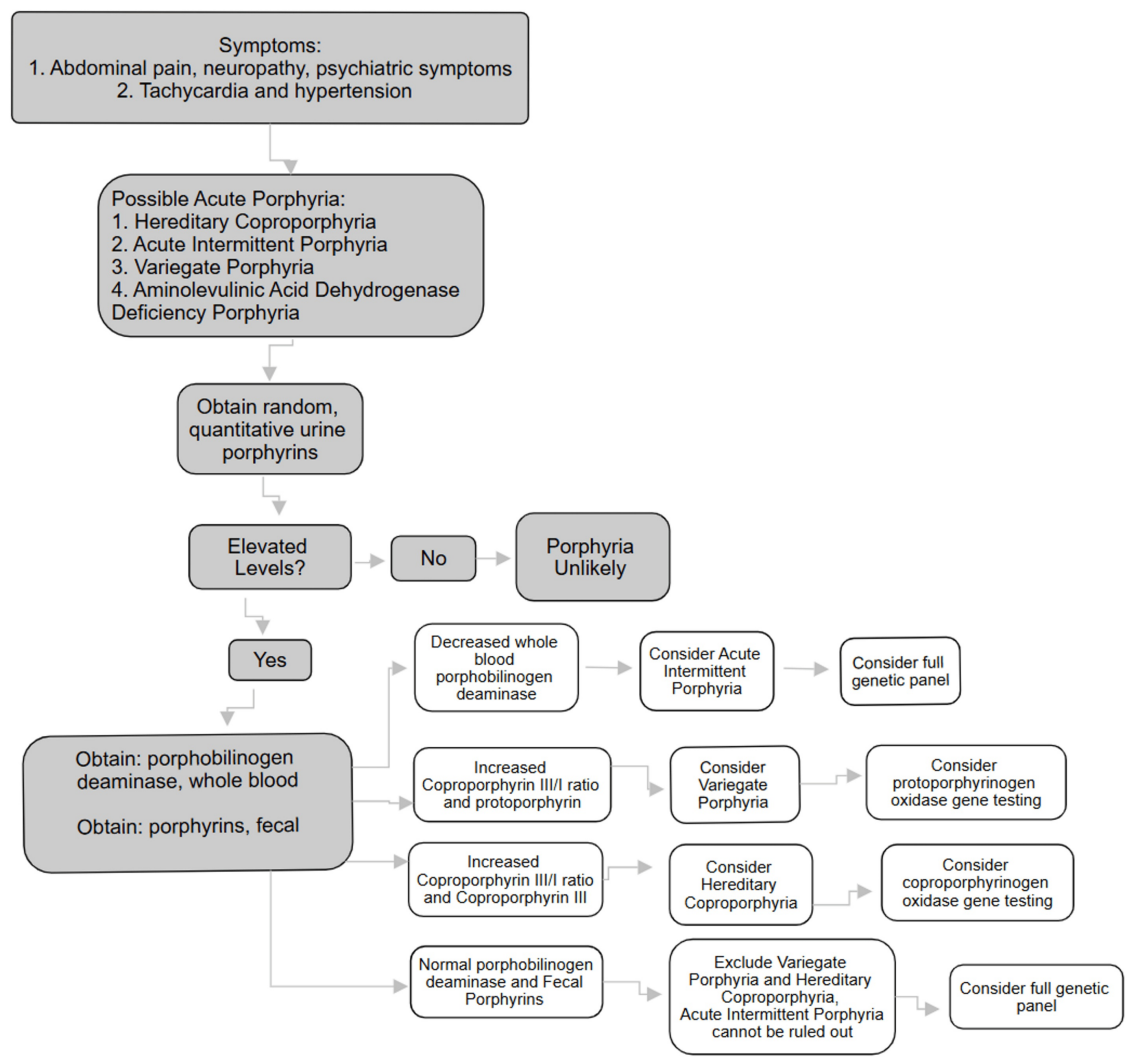
Porphyria Testing Algorithm Image Credits: Lauren Hoffpauir, OMS-III, Author

Once a porphyria illness is suspected, different laboratory tests can be utilized to include or exclude subclasses of porphyria, with genetic testing being the most conclusive [3]. Although considered to be the “gold standard” due to its 97% detection rate of all porphyrias, genetic testing is limited by its expense and accessibility, as there are very few laboratories inside the United States that have the capability to test specifically for the gene that causes porphyria [1]. Random total urine porphyrin testing is a feasible starting point for most patients. Initial urine and fecal porphyria testing are needed to rule out other forms of porphyria and rule in HCP or AIP. The next test should include a fecal porphyria, as a urinalysis resulting in elevated coproporphyrins, or total porphyrin levels is not a sufficient diagnostic for confirming HCP or AIP [4]. This is because many types of porphyrias have similarly elevated and overlapping values, as demonstrated in the flow chart (Figure 1). 

Treatment for porphyria depends on the type of disease present, with a limited range of medications available. Panhematin infusions and Givlaari injections are two options on the market for treating all types of acute porphyrias, which include HCP, AIP, variegate porphyria, and ALA-dehydratase deficient porphyria [1]. Although many experts recommend a high-carbohydrate, low-protein diet for patients suffering from porphyria, it may not be a sufficient remedy for patients with severe attacks. Panhematin is currently the only medication in the U.S. that is able to correct and normalize heme synthesis and porphyrin precursors within the liver [1]. Givlaari is a once-a-month subcutaneous injection that reduced the amount of ALAS1 mRNA, therefore causing a decreased level of ALA and porphobilinogen in the urine [1]. Women taking oral contraceptives should be evaluated to confirm that the estrogen or progesterone is not exacerbating their attacks and to change their birth control to the most appropriate alternative [5]. Some patients, as seen in this patient, may be able to control porphyria attacks by adjusting their diet to include more carbohydrates and fewer proteins. A low-protein diet is thought to decrease the amount of protein available for heme conversion, therefore decreasing the synthesis of porphyrins [6]. In this case, the patient chose the most conservative method of a dietary change and saw a significant decrease in pelvic pain after a three-day implementation of a low-protein diet. 

## Conclusions

Porphyria is difficult to diagnose and can be especially underdiagnosed in young women due to the similarities between gynecological symptoms and porphyrias. Special consideration should be given to women who present with minimal complaints, such as persistent pelvic pain and bouts of forgetfulness. Simple urinalysis and fecal testing are easily obtained, cheap to perform, and can ultimately make a substantial difference for patients suffering from porphyria. It is imperative that samples be taken during an acute flare to allow for accurate diagnosis. Conservative treatments can begin with nominal diet changes to include high-carbohydrate intake and progress to medications if necessary.
